# Adoption of the children’s obesity clinic’s treatment (TCOCT) protocol into another Danish pediatric obesity treatment clinic

**DOI:** 10.1186/s12887-015-0332-9

**Published:** 2015-03-01

**Authors:** Sebastian W Most, Birgitte Højgaard, Grete Teilmann, Jesper Andersen, Mette Valentiner, Michael Gamborg, Jens-Christian Holm

**Affiliations:** The Children’s Obesity Clinic, Department of Pediatrics, Nordsjællands Hospital, Hillerød, Copenhagen University, Dyrehavevej 29, DK-3400 Hillerød, Denmark; Institute of Preventive Medicine, Bispebjerg and Frederiksberg Hospital, The Capital Region, Copenhagen, Denmark; The Children’s Obesity Clinic, Department of Pediatrics, Holbæk University Hospital, Holbæk, Denmark; Institute of Internal Medicine, the Medical Faculty, University of Copenhagen, Copenhagen, Denmark

**Keywords:** Adolescence, BMI, Child, Obesity, Puberty, Treatment

## Abstract

**Background:**

Treating severe childhood obesity has proven difficult with inconsistent treatment results. This study reports the results of the implementation of a childhood obesity chronic care treatment protocol.

**Methods:**

Patients aged 5 to 18 years with a body mass index (BMI) above the 99th percentile for sex and age were eligible for inclusion. At baseline patients’ height, weight, and tanner stages were measured, as well as parents’ socioeconomic status (SES) and family structure. Parental weight and height were self-reported. An individualised treatment plan including numerous advices was developed in collaboration with the patient and the family. Patients’ height and weight were measured at subsequent visits. There were no exclusion criteria.

**Results:**

Three-hundred-thirteen (141 boys) were seen in the clinic in the period of February 2010 to March 2013. At inclusion, the median age of patients was 11.1 years and the median BMI standard deviation score (SDS) was 3.24 in boys and 2.85 in girls. After 1 year of treatment, the mean BMI SDS difference was −0.30 (95% CI: −0.39; −0.21, p < 0.0001) in boys and −0.19 (95% CI: −0.25; −0.13, p < 0.0001) in girls. After 2 years of treatment, the mean BMI SDS difference was −0.40 (95% CI: −0.56; −0.25, p < 0.0001) in boys and −0.24 (95% CI: −0.33; −0.15, p < 0.0001) in girls. During intervention 120 patients stopped treatment. Retention rates were 0.76 (95% CI: 0.71; 0.81) after one year and 0.57 (95% CI: 0.51; 0.63) after two years of treatment. Risk of dropout was independent of baseline characteristics. Median time spent by health care professionals was 4.5 hours per year per patient and the mean visit interval time was 2.7 months. The reductions in BMI SDS were dependent on gender, parental BMI, and family structure in girls, but independent of baseline BMI SDS, age, co-morbidity, SES, pubertal stage, place of referral, hours of treatment per year, and mean visit interval time.

**Conclusions:**

The systematic use of the TCOCT protocol reduced the degree of childhood obesity with acceptable retention rates with a modest time-investment by health professionals.

## Background

Childhood obesity is a growing global epidemic [1,2] and Denmark is no exception [3]. Obesity in childhood is associated with numerous psychosocial [4] and medical complications in the obese child or adolescent, here among increased risk of adulthood obesity [5], coronary heart disease [6], metabolic syndrome [7], as well as risk of cancers [8]. In this respect, it poses a tremendous threat to society by straining the costs of healthcare systems [9] with a negative impact on education and future workforce capabilities.

The health impairments in relation to obesity have led to the recognition of obesity as a disease [10], and underlines why involvement of physicians is essential in treating obesity. There is a crucial need to evaluate clinical management programs that target obese children and adolescents as well as their families. However, treating severe obesity in children and adolescents has proven difficult [11], and treatment results are inconsistent at best [12,13].

Long-term weight loss is significantly harder to obtain than short-term weight loss [11,14] and studies have shown that parental motivation [15], confidence [15], and concomitant parental weight loss [16] are important predictors of success, warranting the use of family-centered chronic care treatment models.

In order to counter childhood obesity, the Department of Pediatrics, Nordsjællands Hospital, Hillerød initiated the Children’s Obesity Clinic in February 2010. The treatment intervention measures were implemented with a few moderations from *The Children’s Obesity Clinic’s Treatment Protocol* (TCOCT) [17], which has produced efficient treatment responses. TCOCT protocol is based on best-practice expert committee recommendations provided by, among others, The American Academy of Pediatrics, The American Medical Association, and the Centers for Disease Control and Prevention [12,18-20], as well as systematic and Cochrane reviews [13,21]. The treatment protocol aims to systematically optimise every aspect of daily life in respect to childhood obesity treatment and prevention of future development of childhood obesity [20]. The primary objective of TCOCT protocol is to help the patient lose weight through individualised behavior-changing techniques targeting both the patient and parents/families as agents of change [12,17,20].

This study aimed to determine changes in body mass indexes (BMI) after individual family-based obesity intervention based on *The Children’s Obesity Clinic’s Treatment Protocol* (TCOCT) developed by Holm and colleagues [17]. Specifically, the objectives are: (1) To evaluate changes in the degree of obesity analysed by changes in BMI SDS during treatment. (2) To explore for associations between baseline BMI SDS and other potential confounders such as age, gender, socioeconomic class (SES), pubertal stage, co-morbidity, place of referral, family structure, hours of treatment per year, mean interval time between visits, and parental BMI. (3) To explore for associations between baseline characteristics, treatment outcomes, and degrees of retention.

## Methods

### Design and setting

This study is a prospective observational study. It included all consecutive patients admitted to *The Children’s Obesity Clinic, Department of Paediatrics, Nordsjællands Hospital, Hillerød,* from February 2010 to March 2013.

### Patients

Patients between 5 and 18 years with a BMI above the 99th percentile for sex and age according to the Danish BMI charts [22] or a BMI rise of more than 5 percentiles in less than 2 years were eligible for referral. No exclusion criteria were used and patients could remain in treatment until the age of 18 or longer if it was deemed necessary. Patients were referred from their general practitioners, school- and community- based doctors, the *Department of Pediatrics*, Nordsjællands Hospital, or from other pediatric departments in the Capital Region of Denmark.

### Data collection

The first visit in the clinic (baseline investigation, 1 hour) was performed by a pediatrician to identify obesity and to screen for underlying causes and possible complications. At baseline, the following variables were obtained: Height was measured by Seca 216 stadiometer, to the nearest millimeter, calibrated monthly by use of a standard-100 cm measure. Weight was measured on a standard calibrated Tanita BC-418 MA to the nearest 0.1 kg without shoes and in light indoor clothing, without need for calibration before 300.000 measurements [23]. Pubertal stage was rated according to Tanner stages. Girls were asked if menarche had occurred. Testicular size was measured by Prader’s orchidometer.

A detailed lifestyle history was provided by the patient and his/her parents in an interview by the pediatrician. The lifestyle history included eating behavior, dietary habits, exercise, transportation patterns, inactivity, bullying, social life, the family’s socioeconomic status, ethnicity, and family structure. Parents BMI was calculated based on self-reported height and weight. Parents were classified as having a normal weight (BMI < 25), being overweight (25 > BMI < 30), or being obese (BMI > 30) according to WHO guidelines [24]. Family structure was classified as follows, by whom the patient was living with: (a) both parents, (b) disrupted family (i.e. single or divorced parents with or without stepfamily), or (c) alternative family structures such as foster families, group homes, or with other family members. The investigation and interview form is available from the authors.

Socioeconomic status (SES) was defined in groups of 1–5 based on the National Statistics Socio-economic Classification [25]. The groups were divided according to occupation as follows: 1. Self-employed, chief executive directors, employees whose work requires skills of the highest educational level. 2. Employees whose work requires skills of a medium long educational level. 3. Trained workers and employees whose work requires skills of a short educational level. 4. Untrained workers, temporary unemployed, and students. 5. People outside the workforce (e.g. senior citizens and disability pensioners). Groups were re-categorised into 1–2: high SES and 3–5: low-medium SES.

To ensure reliable data collection, the clinic’s staff members received one-day training in the use of the TCOCT protocol during a study visit at *The Children’s Obesity Clinic, Department of Pediatrics, Holbæk University Hospital*. Thereafter, all nurses, dieticians, and pediatricians starting in the clinic received training and supervision from more experienced colleagues.

### Treatment intervention

A trusting relationship between the family and the health care personnel was sought established through a structured pedagogical conversation. This strategy sought to optimise diagnosis, treatment, and follow-up of patients and to provide the patient and his/her family with a set of tools in order to implement the needed lifestyle changes. The pediatrician defined a structured and individually tailored treatment plan for the patient in collaboration with the family. The treatment plan was based on the lifestyle history as well as the patient and his/her family’s daily routines such as school and work hours, place of residence, and spare time activities. Any additional underlying disease to obesity, e.g. Prader-Willi syndrome, was integrated in the treatment plan. The plan was delivered in hard copy to support the necessary lifestyle and behavioral changes and to help the patient and his/her family to control the environment. The treatment plan structure offers potentially more than ninety items of advices [17], but individual treatment plans typically contained 15–20 advices at the baseline visit. After the follow-up visits (see below), patients were seen annually by a pediatrician (30 min) to monitor the treatment response and address any necessary adjustments in the treatment plan.

Follow-up visits (45 min) with a trained pediatric nurse were offered in intervals of 8–10 weeks. The follow-up intervals were individualised to the family’s needs and resources (i.e. depending on the patient’s treatment progression, and on practical limitations). Patients were followed by the same nurse to maintain a secure environment for the patient. Regardless of treatment response, families were supported when they showed up at appointments and were reinforced on advices that were integrated in their daily lives. Lack of adherence to intervention advices were specifically identified and discussed and re-implemented, so the treatment plan was revised accordingly. In treatment, all patients were offered one visit (45 min) with a dietician. The dietician would monitor the treatment progression and further specify or modify the diet for the patient. If the family lacked considerable knowledge regarding dieting, food, and cooking, the dietician would offer a second consultation (45 min). Height and weight were measured at all visits by nurses, dieticians, or pediatricians.

Patients were discharged if they missed more than three scheduled appointments and were then categorised as dropouts. Clinical success and hence discharge were decided by the pediatrician using the following criteria: If the patient and the family’s understanding and adherence of the treatment plan were satisfactory and if the patient’s BMI had decreased or stabilised, success was ascertained. The patients were followed until clinical success was achieved, the patients dropped out, the patients moved away, or the patients reached their 25th birthday.

### Statistical methods

All measurements were entered into a Microsoft Access database and exported to Statistical Analysis Software (SAS) for the analyses. Body mass index standard deviation scores (BMI SDS) were determined based on the distribution of a Danish population with the same sex and age [22], using the LMS method. BMI of parents was calculated as weight divided by height squared. The levels of baseline BMI SDS in different groups of patients were compared using ANOVA. The longitudinal development of BMI SDS during treatment was modeled using a generalised linear mixed model. The covariance structure includes a random intercept, allowing each child to have his/her own overall level of BMI SDS, and an exponential residual structure, allowing the covariance between two measurements on the same child to decrease as the time between measurements increases. The mean value of BMI SDS was modeled as a function of time since initiation of treatment, using a cubic spline with three *a priori* -chosen knots (at 2, 11, and 33 months, respectively). The associations between change in obesity and baseline characteristics were assessed by performing a test for interaction between a dichotomised version of the baseline characteristic and time since treatment initiation in the generalised linear mixed model. The degree of retention was illustrated by a Kaplan-Meier plot and by calculating the equivalent retention rates after one and two years. Associations between baseline characteristics and retention were analysed with a cox regression analysis. P-values below 0.05 were considered statistically significant.

### Ethical considerations

This study has been approved by the Danish Data Protection Agency; journal number: 2007-58-0015. All participants gave written consent to participate in treatment. The study is considered as a prospective observational quality development study. Therefore notification to the National Committee on Health Research Ethics or to the National Board of Health was not required [26,27]. All patients received state-funded treatment, as permanent residents in Denmark can use the Danish healthcare system free of charge.

## Results

In the period of February 2010 to March 2013 313 patients (141 boys) were included. Baseline characteristics of patients are shown in Table 1. Table 2 shows the various co-morbidities encountered among the obese patients.

**Table 1:** **Baseline characteristics of included patients**

	**Boys (N = 141)**		**Girls (N = 172)**		**Total (N = 313)**	
	**Median**	**Range**	**Median**	**Range**	**Median**	**Range**
Age (years)	11.9	5.8–17.6	10.6	5.4–17.8	11.1	5.4–17.8
Height (m)	1.56	1.18–1.92	1.48	1.18–1.85		
Weight (kg)	66.2	33.4–166.1	58.8	25.9–129.2		
BMI SDS	3.24	1.31–6.31	2.85	1.4–4.33	3.0	1.31–6.31
Tanner mammae (n = 144)			2	1–5		
Testis (ml) (n = 97)	4	1–25				

N, number of patients; m, meter; kg, kilogram; BMI SDS, body mass index standard deviation score; ml, milliliters.

**Table 2:** **Patients included in the study by referring authority and diagnosis**

	**By the pediatric department (N = 90)**	**By the general practitioner or community-based doctor (N = 223)**
Asthma	13	14
Allergy	19	28
&Psychiatric	10	16
^*^Neurological	13	9
^#^Orthopedic	1	3
^^^Cardiologic	1	2
^@^Endocrine	20	8
Dermatitis	1	3
^+^Abdominal	5	5
“Rare diseases	3	1

&Psychiatric disorders included: autism, personality disorder, self-mutilation; ADHD, attention deficit/hyperactivity disorder; ADD, attention deficit disorder; ^*^Neurological diagnoses included: PCO, polycystic ovary syndrome; Prader-Willi, headache, psychomotor retardation, dyslexia, hearing impairment, non-verbal learning disorder, dyspraxia; CP, cerebral palsy; ^#^Orthopedic diseases included: Legg-Calvé-Perthes syndrome, juvenile idiopathic arthritis, slipped disc; ^^^Cardiologic diseases included: Ventricle-septum defect, sideroblastic anemia, Tetralogy of Fallot,; ^@^Endocrine diseases included: DM, diabetes mellitus; Mb. Addison, gynecomastia, pubertas tarda, growth hormone deficiency; ^+^Abdominal: obstipation, pyelonephritis, lactose intolerance, encoprese; “Rare diseases included: Bardet-Bield syndrome, pituitary tumor, phenylketonuria, factor V leiden-mutation.

At baseline, mean BMI SDS was significantly higher in boys than in girls (boys mean BMI SDS 3.23, range 1.31–6.31, girls mean BMI 2.82, range 1.40–4.33, difference 0.41 SDS (95% CI: 0.25; 0.57, p < 0.0001)). Mean baseline BMI SDS was positively associated with parental BMI: Patients with two overweight parents, or just one obese parent, had a mean of 0.45 (95% CI: 0.25; 0.65, p < 0.0001) BMI SDS higher than patients with just one or no overweight parents. Patients who had parents with low to medium socioeconomic status had a mean 0.35 (95% CI: 0.19; 0.52, p < 0.0001) BMI SDS higher than patients whose parents a high socioeconomic status. Patients referred from general practitioners and school- and community based doctors had a mean 0.20 (95% CI: 0.02; 0.38, p = 0.03) BMI SDS higher than patients referred from the departments of pediatrics. Baseline BMI SDS was significantly associated with puberty in boys, with prepubertal boys having on average 0.55 (95% CI: 0.24; 0.86, p = 0.0007) BMI SDS higher than boys who had entered puberty. This association was not found in girls (p = 0.10). Baseline BMI SDS was independent of age (p = 0.26), family structure (p = 0.46), and co-morbidity (p = 0.81). Median BMI in parents (N = 546) was 28 (range 17.6–53.3), in mothers (N = 272) 27.7 (17.6–53.3) and in fathers (N = 271) 28.4 (18.9–53). In total BMI was <25 in 134 parents (24.5%), 25–30 in 193 (35.3%) and above 30 in 183 parents (33.5%).

**Figure 1:**
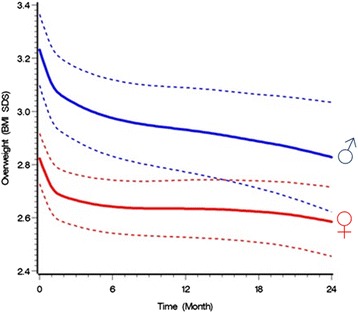
**Mean BMI SDS as a function of time during treatment with 95% confidence intervals in boys (blue) and girls (red) in a chronic care treatment intervention program according to a generalized linear mixed model incorporating all visits from all patients with two or more visits.** BMI SDS, body mass index standard deviation scores. BMI SDS during treatment.

Figure 1 shows the changes in BMI SDS in all children for up to 24 months of treatment in boys and girls. After 1 year of treatment, the mean BMI SDS difference was −0.30 (95% CI: −0.39; −0.21, p < 0.0001) in boys and −0.19 (95% CI: −0.25; −0.13, p < 0.0001) in girls. After 2 years of treatment the mean BMI SDS difference was −0.40 (95% CI: −0.56; −0.25, p < 0.0001) in boys and −0.24 (95% CI: −0.33; −0.15, p < 0.0001) in girls. The proportion of patients achieving a weight loss greater than −0.25 BMI SDS was 41% (95% CI: 34%; 49%) after 1 year of treatment and 53% (95% CI: 43%; 64%) after 2 years. The proportion of patients achieving a weight loss greater than −0.5 BMI SDS was 18% (95% CI: 13%; 24%) after 1 year of treatment and 26% (95% CI: 17%; 36%) after 2 years of treatment.

Patients with one or no overweight parents exhibited a larger decline in BMI SDS with a 0.12 (95% CI: 0.03; 0.21, p = 0.006) BMI SDS lower per year compared to patients with two overweight or at least one obese parent. The reduction was also dependent on gender, with boys attaining a larger decline in BMI SDS with a 0.09 (95% CI: 0.01; 0.17, p = 0.02) BMI SDS lower per year compared to girls. Girls living with both parents attained a larger decline in BMI SDS with a 0.11 (95% CI: 0.01; 0.20 p = 0.03) BMI SDS lower pear year than girls living with a single parent. No association with family structure was found in boys (p = 0.98). Alternative family structures, such as foster families or group homes, were not included for this analysis. The reductions in BMI SDS were independent of age (p = 0.11), baseline BMI SDS (p = 0.51), pubertal stage (p = 0.47 for girls and p = 0.52 for boys), socioeconomic class (p = 0.21), family structure (p = 0.17), co-morbidity (p = 0.65), place of referral (p = 0.56), hours of treatment per year (p = 0.70), and mean visit interval time (p = 0.38).

The mean interval time between visits was 2.7 months (range 0.6–10.2 months), and the median time spent by health care professionals was 4.5 hours per year per patient (range 0.5–25.4 hours).

**Figure 2:**
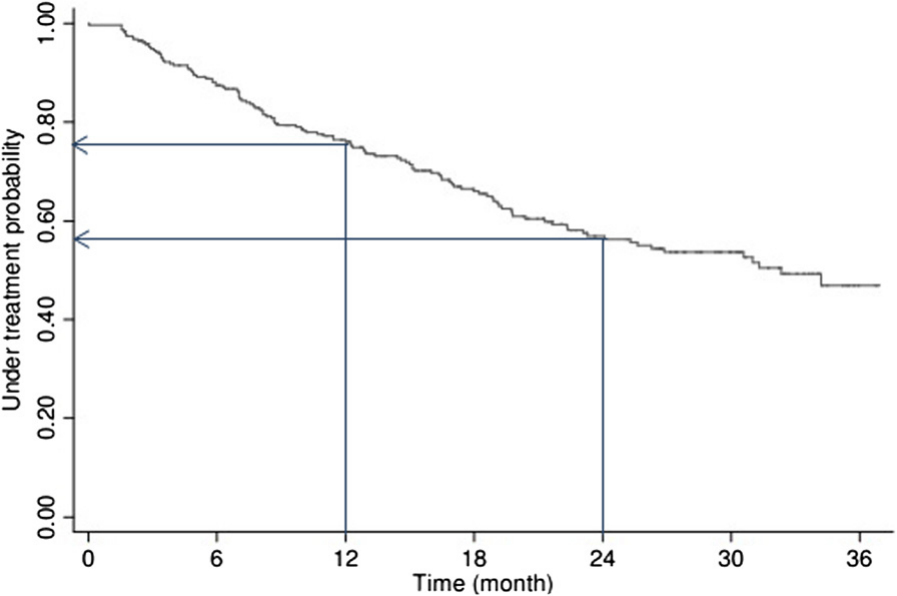
**Kaplan-Meier plot illustrating the rate of dropouts.** An estimated retention function. Rate of drop-outs during treatment.

During intervention 120 patients stopped treatment: 60 (50%) due to families not showing up to appointments: 42 (35%) requested to stop, 16 (13%) stopped because they achieved success, and 2 (2%) dropped out for other reasons. Figure 2 shows the dropout analysis. Retention rates were 0.76 (95% CI: 0.71; 0.81) after one year of treatment, and 0.57 (95% CI: 0.51; 0.63) after two years of treatment. No significant associations between baseline characteristics and retention rates were found. No significant associations were observed between BMI SDS changes during treatment and drop-outs. See Table 3 for hazard ratios.

**Table 3:** **Hazard ratios of dropout in regard to baseline characteristics**

	**Exposed**	**Reference**	**Hazard ratio**	***P*** **value**	**Confidence Interval**
Gender	Girl	Boy	1.10	0.60	0.77; 1.58
Age	Below median	Above median	0.72	0.08	0.51; 1.04
SES group	Group 4 and 5	Group 1, 2, and 3	1.22	0.29	0.85; 1.76
Family structure	Disrupted family	Nuclear family	0.98	0.91	0.66; 1.48
Parental BMI	Obese parents	Non obese parents	1.27	0.29	0.81; 1.99
Co-morbidity	Present	Not present	1.03	0.87	0.71; 1.49
Referral	Pediatric dep.	School- and community based doctors	0.80	0.28	0.53; 1.20
Baseline BMI SDS	Above median	Below median	0.85	0.38	0.59; 1.22
Puberty, boys	Present	Not present	1.79	0.10	0.88; 3.61
Puberty, girls	Present	Not present	0.98	0.94	0.58; 1.65
Longitudinal BMI SDS	Higher than baseline	Lower than baseline	1.38	0.15	0.89; 2.14
Between-visits BMI SDS	Higher than previous visit	Lower than previous visit	1.08	0.72	0.72; 1.63

The hazard ratio compares the risk among the exposed compared to the reference, e.g. girls have a 10% increased risk of dropping out compared to boys.

## Discussion

The Children’s Obesity Clinics Treatment protocol seemed effective in attaining a significant reduction in BMI SDS in severely obese children and adolescents. This reduction was achieved independent of baseline variables such as age, pubertal development, baseline obesity, socioeconomic class, co-morbidity, and place of referral and with a modest investment in manpower. As such our results are consistent with earlier reports evaluating the use of TCOCT [17]. The degree of obesity at baseline was correlated with parental BMI, which is consistent with previous findings [28]. We found no association with age in contrast to previous studies indicating that adolescents are less able to lose weight [29,30], although we did see a trend with patients below median age at baseline dropping out less than the older patients (p = 0.08). Marital disruption has previously been found to have a negative influence on a child’s weight [31,32]. In relation to this, we found that girls, but not boys living with a single parent reduced their weight less successfully compared to living with both parents. We achieved similar results as Holm *et al.* [17], with comparable time spent by health professionals per year and with lower retention rates. No baseline or longitudinal characteristics were significantly associated with an increased risk of dropout. Chronic care treatment is a long process, and while it is more efficient in reaching long-term success [33,34] than short-term treatments are [35], it is also demanding in terms of attendance over time. A high attendance rate has earlier been shown to be an important predictor of success [11], though we found no difference in treatment outcomes between patients who had been seen more frequently in the clinic than others. As this intervention is part of a public hospital setting, standard service was applied to patients and no extraordinary efforts were made to strengthen retention or attendance rates. Nor did we use any exclusion or selection criteria to sort out patients with severe co-morbidities or little motivation. Reinehr *et al*. found a BMI SDS reduction of 0.36 in one year and 0.46 BMI SDS after four years. However, in the latter study only selected motivated families were included and efforts were made to achieve high retention rates, and baseline BMI SDS was 2.46 [29]. Our study population consisted of severely obese patients (median baseline BMI SDS 3.0), and weight loss in this group might be more difficult than in less obese or overweight children [36]. Reductions of more than 0.5 BMI SDS [37,38] and more than 0.25 BMI SDS [39-41] have previously been demonstrated to be a clinically relevant weight loss in terms of improvement in some cardiovascular risk factors and insulin resistance. In the present study BMI SDS was reduced by 0.40 BMI SDS in boys after 2 years of treatment. The proportion of patients achieving a weight loss greater than 0.25 BMI SDS increased over time, reaching 53% after 2 years, suggesting that remaining in treatment is beneficial. Even though we have not reported other measures of success, studies based on previous results from TCOCT protocol reported significant reductions in all fractions of cholesterols (3.8% lower per unit of BMI SDS) [42], lower risk of hyperlipidemia (odds ratio = 0.37 per unit of BMI SDS) [42], and lowered blood pressure [43].

The present study makes a fair representation of the clinical reality in treating severely obese children and adolescents without any prior eligibility criteria and thus includes patients with both other diseases as well as obesity related complications. The reductions in BMI SDS were attained with 4.5 hours per patient per year spent by the clinical personnel. With more than 300 patients and a follow up of more than 2 years in 90 patients, this clinical study provides information about long-term treatment results for obesity. However, as per study design, a control group was not included. In principle, this means that the observed improvements in patient BMI SDS cannot be concluded to be the result of the treatment *per se*, and could be due to other circumstances in the lives of the patients. However, as childhood obesity increases the risk of adulthood obesity [5], a reduction in the degree of obesity in our patients while in treatment seems unlikely to be sporadic or stochastic in its origin. Even so, several other measures such as cholesterol, musclemass/fat free mass, blood pressure and psychological measurements would have provided more specific information about the treatment outcome. Furthermore, we did not follow patients after discharge or dropout, and thus have no data about outcomes after treatment intervention. With 25% dropping out after one year, we cannot neglect a potential dropout bias. Adolescents seem to be the most difficult of patients to target and treat [13]. Other factors we did not measure could have biased treatment outcome, such as patient-family motivation and confidence.

## Conclusion

Larger and longer follow-up studies are needed to provide a further evaluation of the long term results in children and adolescents in treatment for obesity and its complications. Additional measures of success criteria should be identified to provide more accurate information on treatment response and how to treat groups in high-risk of drop out or failure, such as adolescents.

Chronic care treatment of childhood obesity seems to be an efficient treatment intervention which is much needed. The systematic use of the chronic care treatment model TCOCT significantly reduced the degree of obesity in children and adolescents, with only a modest investment in manpower independent of numerous baseline variables. The results were obtained safely and effectively in another hospital setting from where it was originally developed.

## Abbreviations

BMI: Body mass index
CI: Confidence Interval
SDS: Standard deviation scores
SES: Socioeconomic status
TCOCT: The Children’s Obesity Clinic Treatment
